# Hierarchically Structured Polystyrene-Based Surfaces Amplifying Fluorescence Signals: Cytocompatibility with Human Induced Pluripotent Stem Cell

**DOI:** 10.3390/ijms222111943

**Published:** 2021-11-04

**Authors:** Kateřina Skopalová, Katarzyna Anna Radaszkiewicz, Markéta Kadlečková, Jiří Pacherník, Antonín Minařík, Zdenka Capáková, Věra Kašpárková, Aleš Mráček, Eliška Daďová, Petr Humpolíček

**Affiliations:** 1 Centre of Polymer Systems, Tomas Bata University in Zlin, 760 01 Zlin, Czech Republic; skopalova@utb.cz (K.S.); m1_kadleckova@utb.cz (M.K.); capakova@utb.cz (Z.C.); vkasparkova@utb.cz (V.K.); mracek@utb.cz (A.M.); e_dadova@utb.cz (E.D.); 2 Faculty of Science, Masaryk University, 625 00 Brno, Czech Republic; radaszkiewicz@sci.muni.cz; 3 Faculty of Technology, Tomas Bata University in Zlin, 760 01 Zlin, Czech Republic

**Keywords:** biomimetic, surfaces, human-induced pluripotent stem cells, fluorescence signal, cardiomyogenesis

## Abstract

An innovative multi-step phase separation process was used to prepare tissue culture for the polystyrene-based, hierarchically structured substrates, which mimicked in vivo microenvironment and architecture. Macro- (pore area from 3000 to 18,000 µm^2^; roughness (Ra) 7.2 ± 0.1 µm) and meso- (pore area from 50 to 300 µm^2^; Ra 1.1 ± 0.1 µm) structured substrates covered with micro-pores (area around 3 µm^2^) were prepared and characterised. Both types of substrate were suitable for human-induced pluripotent stem cell (hiPSC) cultivation and were found to be beneficial for the induction of cardiomyogenesis in hiPSC. This was confirmed both by the number of promoted proliferated cells and the expressions of specific markers (Nkx2.5, MYH6, MYL2, and MYL7). Moreover, the substrates amplified the fluorescence signal when Ca^2+^ flow was monitored. This property, together with cytocompatibility, make this material especially suitable for in vitro studies of cell/material interactions within tissue-mimicking environments.

## 1. Introduction

Tissue engineering depends on the availability of appropriate cells seeded on appropriate substrates (either 2D or 3D). Human embryonic stem cells (hESC) are considered to be the best lineages due to their ability to develop into any cell type in the human body, and to be grown in vitro and cultivated indefinitely in an undifferentiated state [1]. Their use, however, has aroused much ethical controversy [2]. Currently, it is possible to avoid such controversy by using induced pluripotent stem cells (iPSC) [3,4]. Furthermore, they retain the ability to self-renew and differentiate into any somatic cells [4]. The pioneers in this field, Takahashi and Yamanaka, created iPSCs from mouse fibroblasts in 2006 using viral transduction. They used four factors to induce pluripotency: Oct4, Sox2, c-Myc and Klf4. Surprisingly, the Nanog factor was not needed. The generated mice iPSC was able to differentiate into all germ layers [5]. In 2007, they advanced their research by using the same factors to create iPSCs from human fibroblasts. Pluripotency was verified by the formation of embryoid bodies in vitro, differentiation into neural cells and cardiomyocytes, and the formation of teratoma [6]. 

As mentioned, iPSCs have the potential to create all types of tissues in the human body. The advantage is that the patient’s own cells can be used for therapy and treatment [7] and, therefore, problems with immune response are not relevant. Human iPSCs are able to differentiate into functional cardiomyocytes, thus offering the possibility of their use as autologous cells for the treatment of cardiovascular diseases [8,9]. This is especially important since adult cardiomyocytes have only a limited proliferative capacity, which is not sufficient to repair tissue if the heart is damaged by myocardial infarction. Therefore, iPSC-derived cardiomyocytes (iPSC-CM) appear to be a suitable and inexhaustible source of cardiovascular tissue regeneration [10]. One of the first studies confirming the efficacy of iPSC in the treatment of acute infarction was performed by Nelson et al. The induced cells were able to generate de novo cardiovascular tissue in post-ischemic adult myocardium in vivo [11].

Cells are only one important aspect of tissue engineering. Another is the kind of substrate on which the cells grow. Though substrates can either be flat or three-dimensional (scaffolds), their surfaces remain a crucial feature with respect to cytocompatibility. In this context, only limited information is available on the impact of surface properties and structures on hiPSC behavior. It is known, however, that the structural properties of biomaterials influence the proliferation and maturation of cardiomyocytes [12,13]. Data regarding in vitro cultures of human iPSC-CM (hiPSC-CM) are not very consistent, as it is difficult to mimic the natural niche of the myocardium. In addition, hiPSC-CMs are phenotypically immature compared to adult cardiac cells [12]. hiPSC-CMs are mostly round, mononucleated, and randomly arranged. Native adult cardiomyocytes, on the other hand, are rod-shaped, multinucleated, and arranged in an anisotropic structure [13]. Modifying the topography of material surfaces can contribute to the proper development of hiPSC-CM. For example, Carson et al. [12] created a material mimicking the basement membrane of the myocardium from poly(urethane acrylate). Using lithography, they further created nanopatterned substrates with groove widths ranging from 350 to 2000 nm. Better cardiomyocyte development was achieved on grooves ranging from 700 to 1000 nm. Similarly, Xu et al. [13] created polydimethylsiloxane (PDMS) microchips with a three-dimensional structure resembling an onion epithelium, which facilitated the alignment and elongation of hiPSC-CM. The substrates formed in this way promoted the maturation of cardiomyocytes. The influence of a hierarchically structured surface at the level of macro/meso/micro topography on the attachment and maturation of hiPSC-CM has not been previously investigated.

## 2. Results and Discussion

### 2.1. Characterization of Hierarchically Structured Substrates

The substrates were characterized by scanning electron microscopy, atomic force microscopy, and contact angle measurement. The results from these analyses provided a comprehensive view of the main parameters influencing cell/material interactions, with respect to substrate architecture and surface energy.

**Figure 1:**
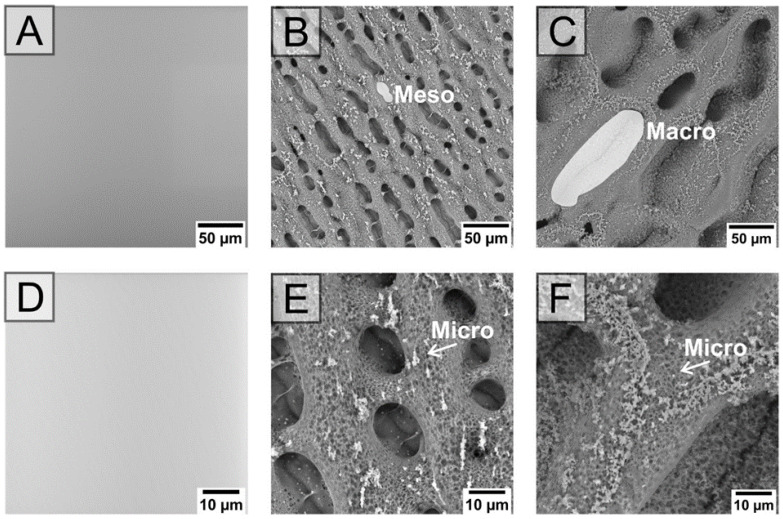
SEM images of native (**A**,**D**), meso-structured (**B**,**E**), and macro-structured (**C**,**F**) substrates. Differences in the sizes of pores are clearly visible from figures (**A**–**C**). Both the hierarchically structured meso- and macro-structured substrates demonstrate similar micro pores.

The SEM images provide a 2D top-down view and clearly show the dimensions of the holes that are available in the cells (see Figure 1). The average pore size was determined to be from 1 µm^2^ for micro- to 300 µm^2^ for meso- or 18,000 µm^2^ for macro-structure (see the Supplementary Materials). The size of pores is not the only property which can be seen in Figure 1. It is also obvious that, although both structured substrates differ at the macro-level, the surfaces are similarly structured on the micro-size levels. The substrates thus provide two cell-instructive properties: the macro-architecture mimicking the 3D dimension normally present in tissues, and the micro-structuring.

**Figure 2:**
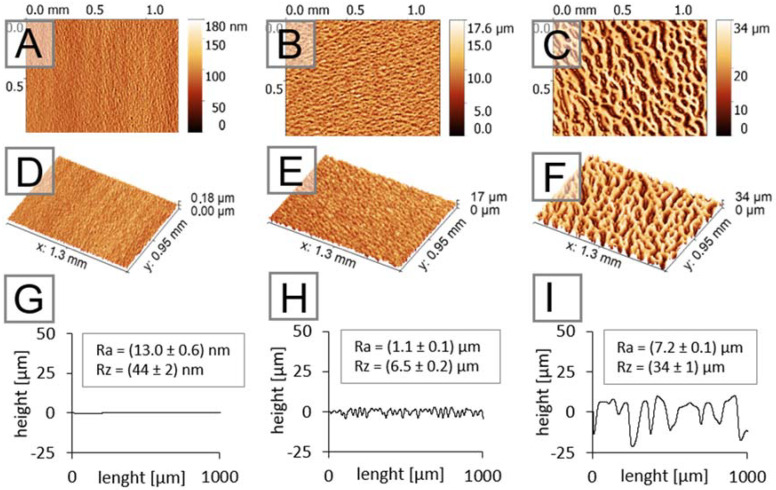
Optical profilometry of native (**A**,**D**,**G**), meso-structured (**B**,**E**,**H**), and macro-structured (**C**,**F**,**I**) substrates. While the native substrate has a roughness in terms of nanometers, the meso- and especially the macro-structured substrates demonstrate roughness in terms of micrometers.

The top-down views provided by SEM, however, do not say anything about the 3D dimensions of substrates, which is a crucial property of the materials presented here, as they can mimic tissue architecture. The 3D images from optical profilometry shown in Figure 2 clearly demonstrate the height differences in the prepared substrates (part H and I of Figure 2) compared to the native tissue culture plastic (part G of Figure 2)—and concretely demonstrates the difference in roughness, which can be considered significant. While the macro-structured substrates exhibited an Ra value of 7.2 ± 0.1 µm, the meso-structured substrates exhibited a value of 1.1 ± 0.1 µm. This provides a more complex view of the 3D architecture, providing the cells with an extracellular signal related to their cytoskeleton organization. The substrate preparation techniques used here also change the surface wettability of native tissue culture polystyrene. The wetting contact angle, representing the most important aspect of the surface related to cell adhesion and the initial stage of cell growth and proliferation, was, therefore, determined (native substrate, 57 ± 2°; meso-structured, 111 ± 3°; macro-structured, 120 ± 3°). However, the modification of surfaces by Matrigel, which is also commonly used for native tissue culture polystyrene when iPSC cells are cultivated, eliminates these initial differences.

### 2.2. Cytocompatibility and Cardiomygenesis of hiPSC

Cells normally obtain important information from their extracellular microenvironment in vivo, especially from the extracellular matrix (ECM). The ECM not only serves as a mechanical support, but also provides an architectural and biochemical cue. The architectural arrangement of the ECM influences the shape of cells, their cytoskeleton and related organelles and structures, while the shape of the cells affects the structure of the ECM [14]. The cells also perceive changes on substrate surfaces from micro- to nano-scales. The creation of structured materials, such as those presented here, gives rise to so-called pseudo 3D models, which, by their structure, will influence the behavior of cells and can be used beneficially, especially for in vitro studies mimicking the natural tissue microenvironment. In this study, the influence of a hierarchically structured surface at the level of macro/meso/micro-topography on the attachment and maturation of hiPSC-CM was investigated.

### 2.3. Proliferation of Undifferentiated hiPSC

**Figure 3:**
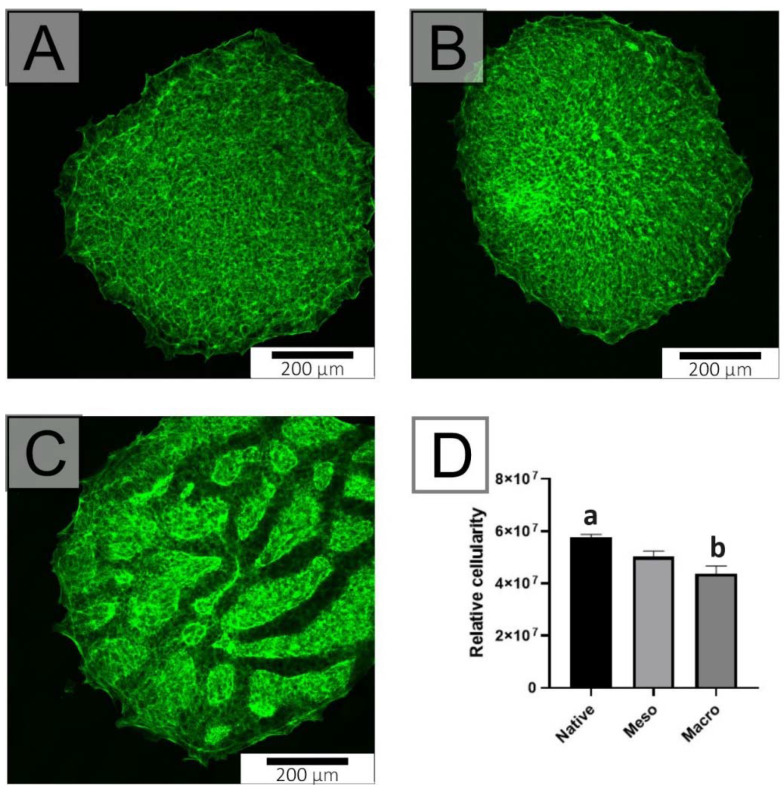
hiPSC cultivated on native (**A**), meso-structured (**B**), and macro-structured (**C**) substrates after four days and counterstained by Phaloidin-FITC. It is clear that all substrates allow cell adhesion and growth. The graph (**D**) shows slightly lower cellularity on the macro-structured substrate four days after seeding. The different superscripts (a/b) express significant differences (*p* ≤ 0.05).

An undifferentiated form of hiPSC was used to determine the cytocompatibility of the substrates. hiPSC lines are unable to adhere and proliferate on substrates without coating the substrate with vitronectin or Matrigel. For this reason, both the native and structured substrates were coated with Matrigel. Figure 3 demonstrates the relative cellularity for each structured substrate compared to the native substrate. A statistically significant difference was observed for the macro-structured substrate, where an inhibition of proliferation was observed compared to control. Although a decrease was noted, it can be concluded from the graph that both samples are suitable for hiPSC culture.

### 2.4. Cardiomyocyte Induction and Maturation

**Figure 4:**
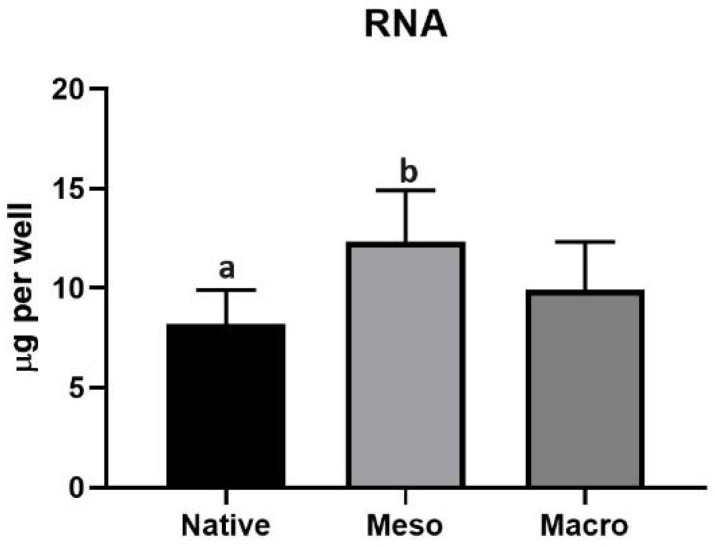
Total amount of RNA isolated from differentiating hiPSCs after 14 days. The different superscripts (a/b) express significant differences (*p* ≤ 0.05) between the number of differentiating cells on the native and meso-porous substrates.

Although hiPSCs can give rise to different cell lines, the methodology used in the present study leads to cardiomyogenesis. The amount of total RNA on the surfaces was determined after 14 days of differentiation. Figure 4 shows that the amount of RNA did not differ substantially between the native substrate and macro-porous substrate. On the other hand, the total RNA isolated from the differentiating cells remained more statistically significantly on the meso-structured than the native substrate.

**Figure 5:**
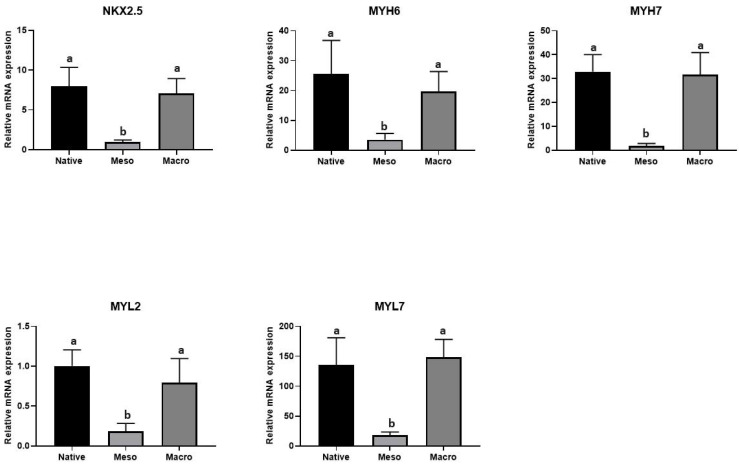
The expressions of markers of cardiomyogenesis detected by qRT PCR. The different superscripts (a/b) express significant differences (*p* ≤ 0.05).

However, it is not possible to determine the proportion of individual species of the cell population from the total amount of isolated RNA. Therefore, the expressions of genes associated with cardiogenesis were evaluated; specifically, the Nkx2.5 [15], MYH6 [16], MYH7 [17], MYL2 [18] and MYL7 [19] genes were selected as representative cardiomarkers. The results presented in Figure 5 indicate the proportion of cells undergoing cardiomyogesis. Although a larger amount of total RNA was isolated on the meso-structured sample than on the macro-structured sample (see Figure 4), the expressions of cardiomyogenesis-related genes showed the opposite tendencies; thus, they were significantly lower on the meso-structured substrate (Figure 4). It follows that the hierarchical topography of meso-structured substrates supports the adhesion of differentiated hiPSCs but does not contribute to the formation of more cardiomyocytes. Higher expressions of specific markers were noted on the macro-structured substrate. Although there were fewer cells on the macro-structured sample, this structure allowed for better cardiomyocyte attachment or had a positive impact on differentiation.

**Figure 6:**
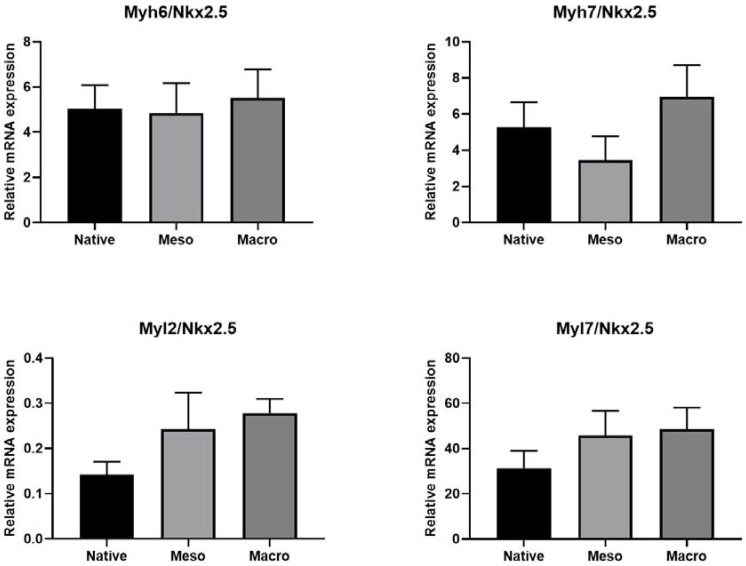
Ratio of gene expression (Myh6, Myh7, Myl2 and Myl7) to reference gene (Nkx2.5). Evidence of mature cardiomyocytes. Cardiomyocyte maturation was not significantly different on meso- and macro-structured substrates. Therefore, there is no need to provide a statistical analysis.

To determine the maturation of cardiomyocytes, the expressions of the Myh6, Myh7, Myl2 and Myl7 genes were related to Nkx2.5, which can be considered as the reference gene for cardiomyocytes [20,21]. The data presented on Figure 6, however, are only related to the cardiomyocyte population (whereas Figure 5 shows gene expression in all cells present on the substrate). It is clear that there were no major differences in cardiomyocyte maturation. Thus, it can be concluded that none of the structured surfaces inhibited cardiomyocyte maturation. Therefore, the different topographies only affected their attachment or differentiation. These findings will be the subject of further investigation in the context of cardiomyogenesis on structured surfaces. 

In conclusion, it can be stated that both hierarchically arranged topographies are suitable for hiPSC cultivation. Meso-structured and macro-structured structures had no inhibitory effect on cardiomyocyte maturation. By expressing specific markers (Myh6, Myh7, Myl2, Myl7), it was found that cardiomyocytes adhered better to the macro-structured substrate. The main difference between the individual samples was the size of the pores. The results suggest that hiPSC-derived cardiomyocytes (hiPSC-CM) attach better to macro-structured substrates.

### 2.5. Structured Surface Amplified Fluorescence Signal

One possible method of determining the presence of mature cardiomyocytes is to observe Ca^2+^ flow. This highly specific and intricate regulatory system develops gradually, with a progressive maturation of specialized structures and an increase in the capacity of Ca^2+^ sources and sinks [22]. Remarkably, through this analysis, extraordinary properties were determined for structured substrates.

**Figure 7:**
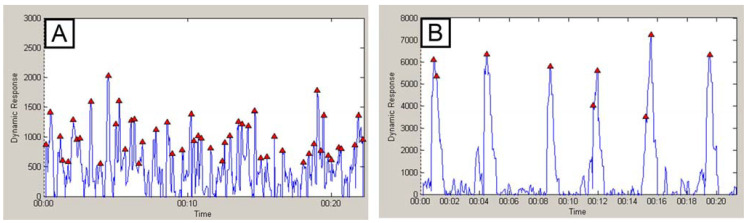
Representative records of the Ca^2+^ intracellular wave in beating cardiomyocyte determined by an Fura4 probe. Record of hiPSC-CM growing on native (**A**) and macro-structured (**B**) surfaces. HiPSC-CM photos and Ca^2+^ waves were computed from the camera record. The fluorescence microscope was fitted for green fluorescence.

The modification of the tissue culture plastic used here led to the amplification of the fluorescence signal, as seen in Figure 7. The evidence of an amplified signal is also clear from the attached video (see the Supplementary Materials). The modified substrates (based on common tissue culture plastic) can be used to increase the sensitivity of the Ca^2+^ transition analysis of contracting cardiomyocytes, as observed in our experiments. This means that it is possible to use a normal camera for recording signals or a low concentration of Ca^2+^ sensitive fluorescent probes (Fura4 here), and thus decrease the phototoxicity of the assay. As shown in the attached video, the hiPSC-CM have the same beating frequency independent of the used substrate. Nevertheless, the analysis of intracellular Ca^2+^ management in contracting hiPSC-CM is more easily performed on structured substrates, due to their higher reflectivity. The Ca^2+^/Fura4 signal on the native substrate was close to the background signal, and individual Ca^2+^ waves were undetectable when a normal camera was used. In contrast, the signal was significantly amplified for hiPSC-CM growing on structured substrates, where individual Ca^2+^ waves were clearly recognizable (sees Supplementary Materials Video).

## 3. Materials and Methods

### 3.1. Materials and Reagents

As an initial substrate, tissue culture polystyrene (PS) Petri dishes with a diameter of 34 mm, sterilized by UV radiation and free from pyrogens and DNA/RNA (TPP Techno Plastic Products AG, Trasadingen, Switzerland), were used (further highlighted as “native” substrate). Ultrapure water (18.2 MΩ.cm), tetrahydrofuran (THF, suitable for high-performance liquid chromatography (HPLC grade); Sigma-Aldrich Ltd., Gillingham, UK), and 2-ethoxyethanol (ETH, p.a.; Sigma-Aldrich Ltd., Gillingham, UK) were used.

### 3.2. Preparation and Characterization of Hierarchically Structured Substrates

Solution preparation: For the surface modification, three types of mixture were prepared. Mixture 1 consists of THF and ETH in a volume ratio of 1:2. Mixture 2–PS, THF, and ETH in a ratio of 2.68 g:10 mL:20 mL–was prepared by dissolving PS in THF for 3 h at room temperature. Then, the ETH was added dropwise while constantly stirring the mixture. To prepare Mixture 3, THF and ultrapure H_2_O were mixed in a volume ratio of 5.6:4.4.

Surface modification: All surfaces were modified by deposition of solvent mixtures using a homemade spin coater [23]. Adjustable parameters include the dosed volume, the number of doses, the delay between each dose, the speed of rotation, and the time of rotation of the sample after the last dose. For the deposition of solvent mixtures, a glass syringe with a metal needle placed 30 mm above the center of the native substrate was used. All modification processes were performed at 25 °C under 50% air humidity, as described in our previous works [23,24]. The resulting substrates can be classified as meso-structured (from 10 to 50 µm) or macro-structured (above 50 µm) and micro-structured (from 0.1 to 10 µm) according to the size of irregularities.

Optical Goniometry: The static contact angles of water were measured and evaluated on all substrates before and after the plasma modification process by means of a DSA30 automated drop shape analyzer and Advance software (KRÜSS GmbH, Hamburg, Germany). The measurements were performed as follows: 5 drops of ultrapure water, each with a volume of 3 μL, were deposited on the measured surface at room temperature and at 25 °C under 50% humidity. 

Scanning Electron Microscopy (SEM): Substrate surfaces were observed by means of a Phenom Pro scanning electron microscope (Phenom-World BV, Eindhoven, Netherlands). Samples were analyzed at an acceleration voltage of 10 kV in backscattered electron mode, with the magnification ranging from 1000× to 4000×. Measurements were carried out on samples without prior metallization, using a holder allowing for the reduction in charges on polymeric materials.

Optical profilometry: Surface topographies were characterized by a 3D optical microscope Contour GT-K (Bruker, MA, USA) based on white light interferometry with the use of 20× objective lenses. The resulting 2D and 3D topography maps were processed in the Gwyddion 2.55 software.

Contact profilometry: Changes in the surface topography and roughness of all substrates were characterized by a DektaXT contact profilometer (Bruker, MA, USA). A tip with a curvature radius of 12 µm and a pressure equivalent to 5 mg was used. The evaluation of surface roughness values (Ra) and maximum height changes (Rz) was determined from 5 individual measurements according to the SME B46.1 standard.

Image processing and analysis: Images from SEM were processed using ImageJ software, version 1.5 (W. Rasband, National Institutes of Health, Bethesda, MD, USA); the scale bars were added.

### 3.3. Cytocompatibility and Cardiomygenesis of hiPSC

Cell lines: The cytocompatibilities of the substrates were determined using the human-induced pluripotent stem cell (hiPSC) line [25]. 

Cultivation protocol: The hiPSC line was cultivated on substrates coated with Vitronectin (0.5 μg/cm^2^; passage and self-renewal) or with Matrigel (18−20 μg/cm^2^; differentiation/cardiomyogenesis). In all experiments, the cells (404 cells per cm^2^; 2 mL of cell suspension per modified Petri dish (35 mm in diameter)) were seeded as small cell clumps, each containing approximately 3–10 cells. In all the aforementioned experiments, the cells were cultured in 2 mL media per well on modified Petri dishes. The following assays were performed. First, the initial growth/proliferation of cells was determined by measuring the level of ATP in cell lysate 4 days after seeding [26]. Next, the degree of cardiomyogenesis in hiPSC populations, based on the expressions of transcripts related to the cardiomyocyte phenotype, was evaluated after 14 days of cell differentiation (Table 1) [25].

Gene expression: Total RNA was extracted by RNeasy Mini Kit (Qiagen, Hilden, Germany). Complementary DNA was synthesised using M-MLV reverse transcriptase kit (Sigma-Aldrich) following the manufacturer’s instructions. qRT-PCR was performed in a Roche Light-cycler (Roche, Basel, Switzerand). HPRT1 was used as a reference gene, and primer sequences and probes are listed in Table 1 [25].

Analysis of Ca^2+^ transients in beating cardiomyocytes: Cardiomyocytes were washed with Tyrode solution consisting of 135 mM NaCl, 5.4 mM KCl, 10 mM HEPES, 0.33 mM NaH_2_PO_4_, 0.9 mM MgCl_2_, 0.9 mM CaCl_2_, and 10 mM glucose (pH 7.4 adjusted with NaOH). Cells were loaded with 10 mM Fluo-4 AM dye (Life Technologies–Molecular Probes, F14201) in the dark at 37 °C for 45 min. Following dye loading, the cells were washed with Tyrode solution and further incubated for 45 min at 37 °C. Imaging was performed on an Olympus IX-51 inverted fluorescence microscope with an Olympus E-450 camera. The beating frequency and the width of individual peaks of Ca^2+^ transients were analysed from recordings by means of a CB Analyser, as described previously [27]. 

Quantitative analysis of fluorescence: The a375 melanoma cell line, which constitutively expresses green fluorescent protein (GFP), was used to quantitatively analyse the fluorescence signal [28]. Cells were seeded on both standard and modified tissue culture plastic in DMEM media with 10 % fetal bovine serum, 100 UI/mL penicillin, and 0.1 mg/mL streptomycin. Cells were cultivated in standard conditions at 37 °C in a humidified atmosphere containing 5 % CO_2_. Fluorescence signals were recorded using an Olympus BX51 fluorescence microscope equipped with an Olympus XM10 digital camera that was set to the same exposure time for all samples. The obtained data were analysed and quantified using ImageJ software.

Statistical analysis: Prior to ANOVA, the normal distribution of data was verified, and deviant values were determined by Dixon’s Q test. Statistical significance was determined by ANOVA with post hoc Tukey’s Multiple Comparison Test. More detailed information and values of significance are provided in the description of individual results.

## 4. Conclusions

Common in vitro experimental conditions do not mimic the in-vivo-occurring 3D architecture. The experiments thus lack these important external cell-instructive cues. The tissue culture, polystyrene-based substrates were, therefore, modified by the sequential dosing of solution mixtures on rotating surfaces to form a hierarchically arranged structure (micro-, meso and macro-structure). The substrates prepared here mimic the in vivo microenvironment. Structured substrates were tested in the context of their impact on the cardiomyogenesis of human-induced stem cells. Both types of substrate were suitable for culturing induced cells and, in addition, were found not to inhibit the maturation of cardiomyocytes. Furthermore, while monitoring the Ca^2+^ flow in mature cardiomyocytes, substrates were found to amplify the fluorescence signal, offering improved monitoring options when observed via a normal camera for recording signals, or via a low-concentration reading of Ca^2+^ sensitive fluorescent probes, and thus decreasing the phototoxicity of the assay. The substrates prepared here offer a new possibility for the experimental set-up of in vitro experiments.

## Figures and Tables

**Table 1:** Primer sequences for target and reference genes used in qRT-PCR assays; F = forward primer (5′-3′), R = reverse primer (3′-5′).

NCBI Reference Sequence Gene	Primer Sequence	Tanealing (°C)	UPL Probe No.
NM_004387.3 NKX2.5	F cacctcaacagctccctga R ctaggtctccgcaggagtga	59 60	#7
NM_021223.2 MYL7	F gggtggtgaacaaggatgag R gtgtcagggcgaacatctg	60 60	#2
NM_000432.3 MYL2	F gcaggcggagaggttttc R agttgccagtcacgtcagg	60 60	#63
NM_000257.3 MYH7	F catctcccaaggagagacca R ccagcacatcaaaagcgtta	60 59	#73
NM_002471.3 MYH6	F ctcaagctcatggccactct R gcctcctttgcttttaccact	60 59	#63
NM_000194.2 HPRT1	F tgaccttgatttattttgcatacc R cgagcaagacgttcagtcct	59 60	#73

## Data Availability

Data are available from correspondent authors.

## Supplementary Materials

The following are available online at https://www.mdpi.com/article/10.3390/ijms222111943/s1.

## Abbreviations

ECM	extracellular matrix
ETH	2-ethoxyethanol
hESC	human embryonic stem cells
iPSC	induced pluripotent stem cells
iPSC-CM	iPSC-derived cardiomyocytes
PDMS	polydimethylsiloxane
Ra	roughness
SEM	scanning electron microscopy
THF	tetrahydrofuran
