# Supplementary material for: Hierarchically Structured Polystyrene-Based Surfaces Amplifying Fluorescence Signals: Cytocompatibility with Human Induced Pluripotent Stem Cell
Source: Int J Mol Sci. 2021 Nov 4;22(21):11943. doi: 10.3390/ijms222111943 (PMC8584612; doi:10.3390/ijms222111943)
Supplement: Supplementary file 1 [file ijms-22-11943-s001.zip › Supplementary_data..pdf]

International Journal of *Molecular Sciences*

Supplementary information to article:

Hierarchically structured polystyren-based surfaces amplifying fluorescence signals: cytocompatibility with human induced pluripotent stem cell

Kateřina Skopalová[1], Katarzyna Anna Radaszkiewiczb[2], Petr Humpolíček [1,3,*], Markéta Kadlečkováa[1,3], Jiří Pacherník[2*], Antonín Minařík[1,3*], Zdenka Capáková[1], Věra Kašpárková[1,3], Aleš Mráček[1,3], Eliška Daďová[1]

[1] Centre of Polymer Systems, Tomas Bata University in Zlin, 760 01 Zlin, Czech Republic
[2] Masaryk University, Faculty of Science, 625 00 Brno, Czech Republic
[3] Faculty of Technology, Tomas Bata University in Zlin, 760 01 Zlin, Czech Republic

Corresponding authors: E-mail: humpolicek@utb.cz, E-mail: jipa@sci.muni.cz , E-mail: minarik@utb.cz

Received: date; Accepted: date; Published: date

1. Homemade spin coater

All surfaces were modified by deposition of solvent mixtures using a homemade spin coater (Figure 1) [1].

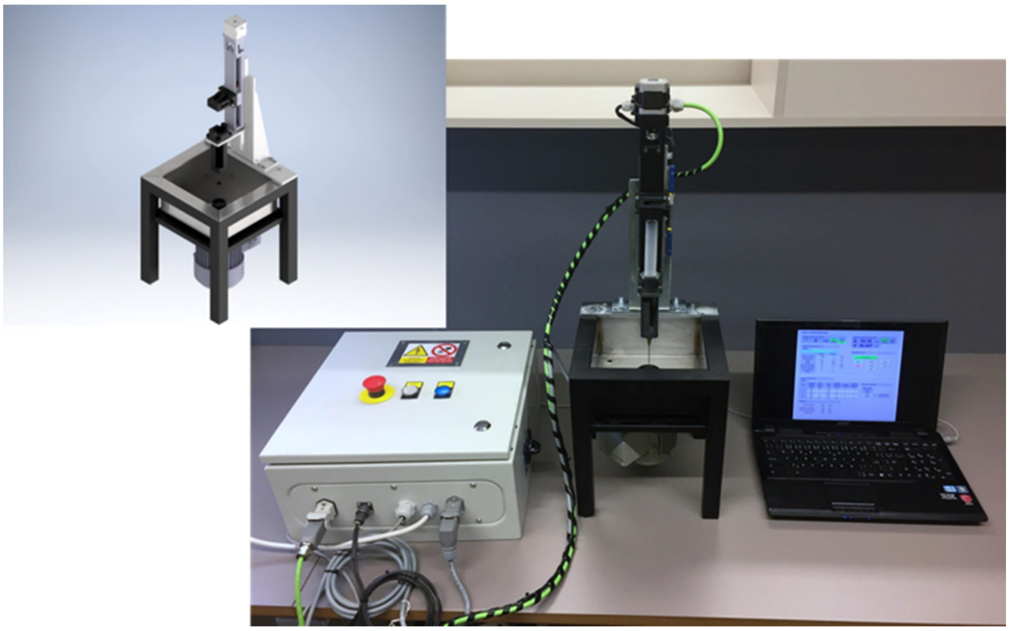

Figure S1: Equipment for modification of surface topography of polymers. On the right, a set of devices with control electronics for precise dosing of mixed solutions on rotating material. Top left, a preview on the functional part of the device.

2. Image analysis of surface pore area

Data obtained by 3D optical microscope (Contour GT-K, Bruker) were processed using the the Gwyddion – Free SPM data analysis software, version 2.55 (D. Nečas, P. Klapetek, Czech Metrology Institute, Czech Republic) and ImageJ version 1.5 (W. Rasband, National Institutes of Health, United States).

a) Cross sections of the 3D topography maps were obtained by Profile Tool in Gwyddion software. For macro structured surfaces the cross section was taken in the height of 18 µm (z axis) and for meso structured the height of cross section was 5 µm (z axis). These resulting 2D images were used to analyze pores area and number of pores for a given area (800x800 µm) in ImageJ software.

b) In the ImageJ software the 2D images of crossed sections were contrasted to highlight the pores (Figure 2 - A, C). Than the function "analyze particles" was used to get the images of outlined and numbered pores (Figure 2 - B, D) and table of results which contained information about number of pores and its area distribution. Resulting data were processed to the form of histogram (Figure 2).

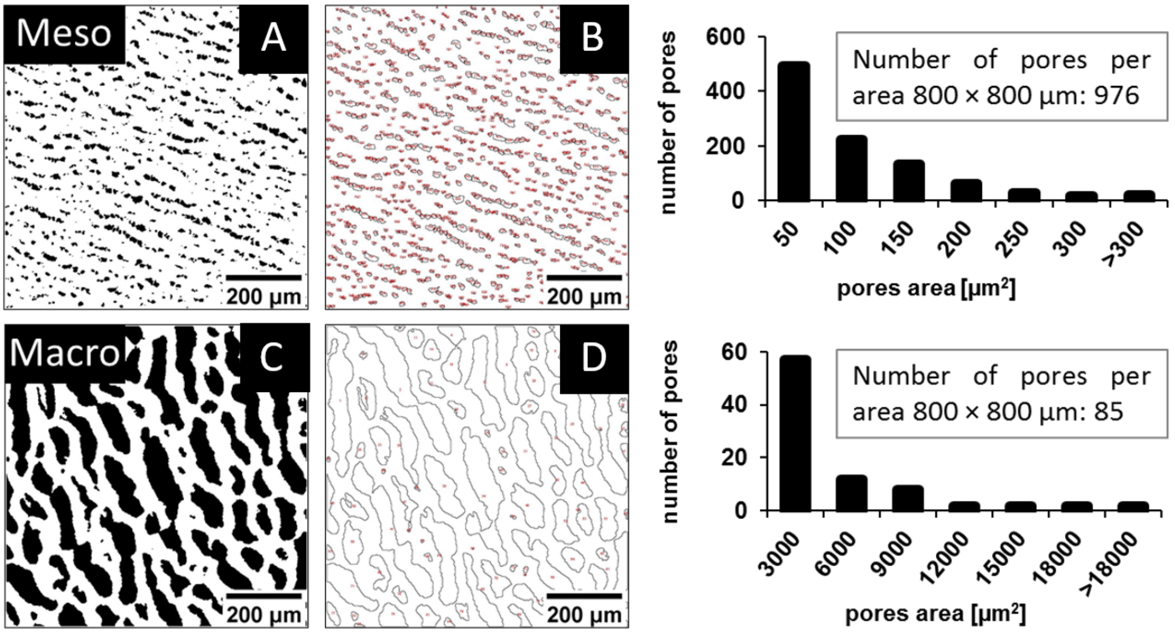

Figure S2: 2D images of cross sections with highlighted pores (black areas) A - for meso) and C - for macro structured surfaces. Images B – for meso and D – for macro structured surfaces shows resulting outlined and numbered pores.

3. Fluorescence signal amplification

To determine which type of surface topography has a major effect on the amplification of the fluorescent signal, a number of samples were prepared, Fig. 3a:

A) **Native** - tissue culture polystyrene (PS) Petri dishes with a diameter 34 mm, sterilized by radiation and free from pyrogens and DNA/RNA (TPP Techno Plastic Products AG)

B) **Micro** - tissue culture PS Petri dishes modified at TSSC equipment [1,2] by dosing of 5x200 µl of THF:ETH in volume ratio 7:3 in time interval 5 s at 23 °C, 2200 rpm.

C) **Meso** - tissue culture PS Petri dishes modified at TSSC equipment [1,2] by dosing of 5x200 µl of THF:ETH in volume ratio 1,5:8,5 in time interval 5 s at 23 °C, 2200 rpm.

D) **Macro** - tissue culture PS Petri dishes modified at TSSC equipment [1,2] by dosing of 3x300 µl of THF:H2O (5,6:4,4) in time interval 5 s at 23 °C, 1500 rpm.

E) **Meso/micro** - tissue culture PS Petri dishes modified at TSSC equipment [1,2] by dosing of 1x300 µl of THF:ETH in volume ratio 1:2 at 23 °C, 1500 rpm.

F) **Macro/micro** – Modification step 1 - tissue culture PS Petri dishes modified at TSSC equipment [1,2] by dosing of 3x300 l of THF:H2O in volume ratio 5,6:4,4 in time interval 5 s at 23 °C, 1500 rpm. Modification step 2 - macro structured PS Petri dishes prepared in the first step modified at TSSC equipment [1,2] by dosing of 1x300 µl of THF:ETH in volume ratio 1:2 at 23°C, 1500 rpm

For the purposes of this work the surface irregularities (pores) are refered to as native (under 0,1 µm), Micro (from 0,1 to 10 µm), Meso (from 10 to 50 µm) and Macro (over 50 µm).

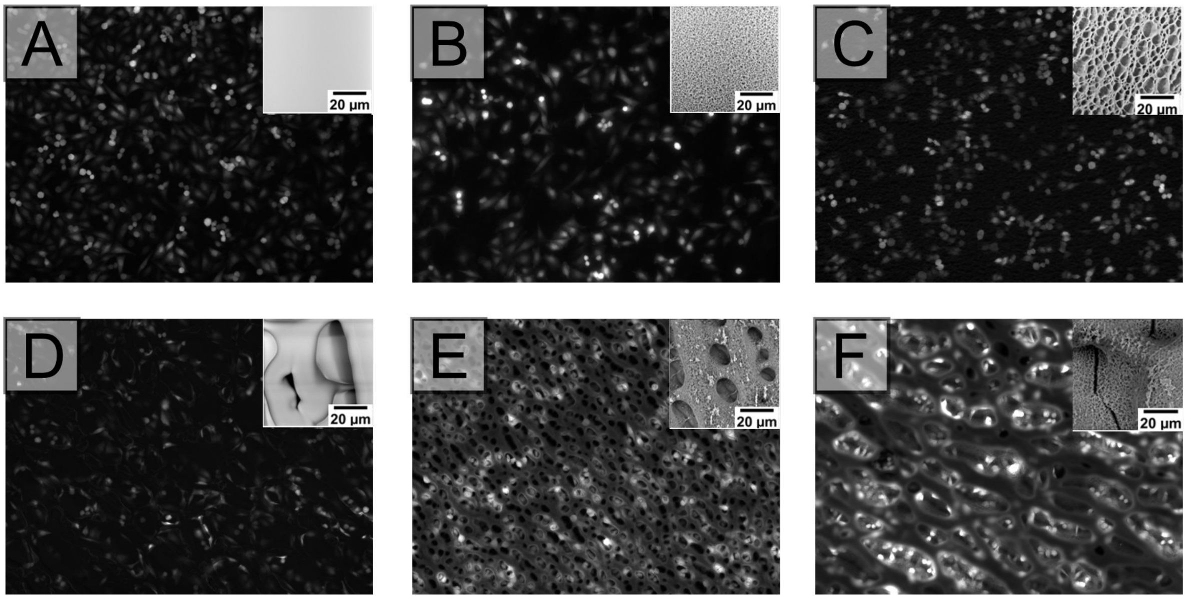

Figure S3a: The image of fluorescence signal and SEM images of the native (A), micro (B), meso (C), macro (D) meso/micro (E), and macro/micro (F) substrates.

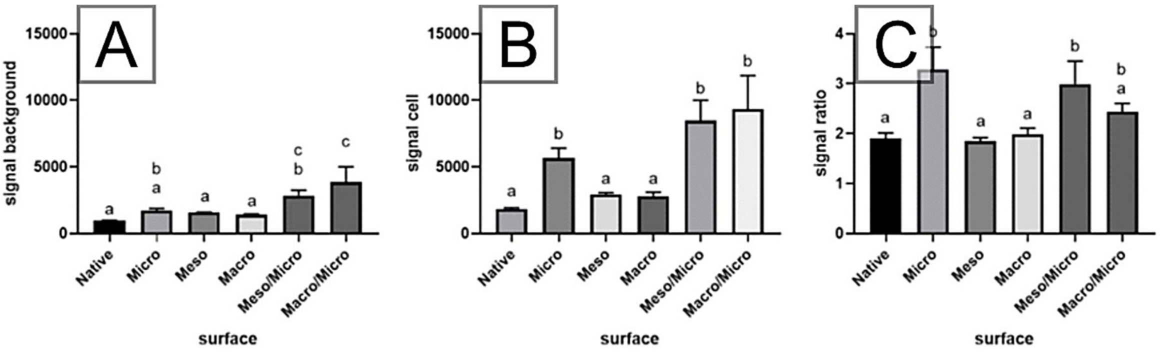

Figure S3b: The magnitude of the fluorescence signal recorded on A) the autofluorescence of the surfaces and the B) fluorescence of the labeled cells, C) the ratio of the signals shown in B) and C). The different superscripts express significant differences (p≤0.05).

To confirm the amplification of the fluorescence signal by structured substrates, the a375 melanoma cell line continuously expressing GFP [3] was used. From the evaluation of fluorescence signals on differently structured surfaces, Fig. 3, these conclusions follows: Structured PS surfaces have a much higher autofluorescence signal than smooth ones, Fig. 3b part A. This fact is most likely due to the multiple reflection of both excitation and emission radiation on differently structured surfaces. As well we can observe a much stronger fluorescence signal in the case of labeled cells on structured surfaces, Fig. 3b part B. The signal amplification is greatest on surfaces characterized by micro-irregularities with a diameter in the range of 0.1 to 10 μm, as shown by the fluorescence signals ration in Fig. 3b part C (micro surface). This means that for the largest quantum yields of the fluorescence process are responsible micro inequalities, which in combination with meso and macro pores create a new type of highly effective surface for the study of cellular processes.

4. The video of flurescence signal detected on native and macro-structured substrates.

References

1. Wrzecionko, E.; Minařík, A.; Smolka, P.; Minařík, M.; Humpolíček, P.; Rejmontová, P.; Mráček, A.; Minaříková, M.; Gřundělová, L., Variations of Polymer Porous Surface Structures via the Time-Sequenced Dosing of Mixed Solvents. ACS Applied Materials & Interfaces 2017, 9 (7), pp 6472–6481, doi: https://doi.org/10.1021/acsami.6b15774.

2. Minařík, M.; Wrzecionko, E. Minařík, A.; Grulich, O.; Smolka, P.; Musilová, L.; Junkar, I.; Primc, G.; Ptošková, B.; Mozetič, M.; Mráček, A., Preparation of Hierarchically Structured Polystyrene Surfaces with Superhydrophobic Properties by Plasma-Assisted Fluorination. Coatings 2019, 9 (3), doi: https://doi.org/10.3390/coatings9030201.

3. Radaszkiewicz, T.; Nosková, M.; Gömöryová, K.; Vondálová Blanářová, O.; Radaszkiewicz, K. A.; Picková, M.; Víchová, R.; Gybel', T.; Kaiser, K.; Demková, L.; Kučerová, L.; Potěšil, D.; Zdráhal, Z.; Souček, K.; Bryja, V., RNF43 inhibits WNT5A driven signaling and suppresses melanoma invasion. bioRxiv 2021, doi: https://doi.org/10.1101/2021.02.08.430210.

Publisher's Note: MDPI stays neutral with regard to jurisdictional claims in published maps and institutional affiliations.

© 2020 by the authors. Submitted for possible open access publication under the terms and conditions of the Creative Commons Attribution (CC BY) license (http://creativecommons.org/licenses/by/4.0/).
